# [1-(3-Nitro­phen­yl)-2,3-dihydro-1*H*-naphtho[1,2-*e*][1,3]oxazin-3-ylidine]malonaldehyde

**DOI:** 10.1107/S1600536809006023

**Published:** 2009-02-25

**Authors:** M. NizamMohideen, A. SubbiahPandi, N. Panneer Selvam, P. T. Perumal, M. Damodiran

**Affiliations:** a Department of Physics, The New College (Autonomous), Chennai 600 014, India; b Department of Physics, Presidency College (Autonomous), Chennai 600 005, India; c Organic Chemistry Division, Central Leather Research Institute, Chennai 600 020, India

## Abstract

The oxazine ring in the title compound, C_21_H_14_N_2_O_5_, adopts a flattened boat conformation. The nitro­phenyl ring and the naphthalene ring system enclose a dihedral angle of 89.2 (1)°. An intra­molecular hydrogen bond is formed between the NH group and one of the adjacent carbonyl O atoms. In addition, the NH group forms an inter­molecular hydrogen bond to a symmetry equivalent of this carbonyl O atom, connecting the mol­ecules into centrosymmetric dimers. The structure also contains C—H⋯O inter­molecular inter­actions.

## Related literature

For hydrogen-bond motifs, see: Bernstein *et al.* (1995 ▶). For ring puckering parameters, see: Cremer & Pople (1975 ▶); Nardelli (1983 ▶).
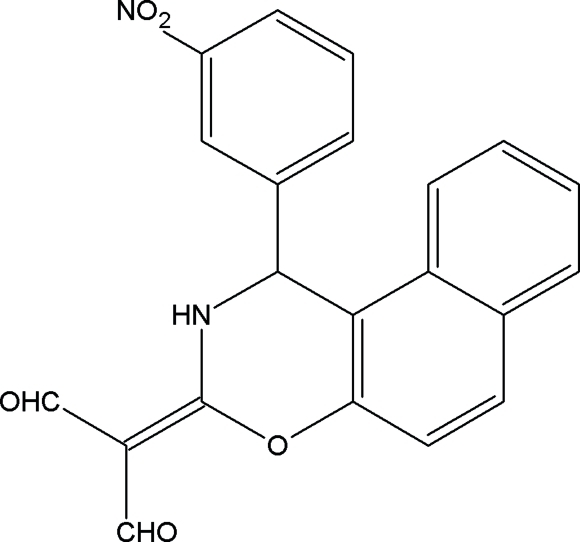


## Experimental

### 

#### Crystal data

C_21_H_14_N_2_O_5_
*M* *_r_* = 374.34
Monoclinic, 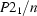

*a* = 7.9191 (4) Å
*b* = 23.3876 (11) Å
*c* = 9.6199 (5) Å
β = 104.632 (2)°
*V* = 1723.91 (15) Å^3^
*Z* = 4
Mo *K*α radiation
μ = 0.11 mm^−1^
*T* = 293 K
0.33 × 0.27 × 0.25 mm

#### Data collection

’Bruker Kappa APEXII CCD diffractometer’
Absorption correction: multi-scan (*SADABS*; Bruker, 2004 ▶) *T*_min_ = 0.897, *T*_max_ = 0.974
16263 measured reflections
3023 independent reflections
2334 reflections with *I* > 2σ(*I*)
*R*_int_ = 0.032

#### Refinement

*R*[*F* ^2^ > 2σ(*F* ^2^)] = 0.036
*wR*(*F* ^2^) = 0.102
*S* = 1.00
3023 reflections
253 parameters
H-atom parameters constrained
Δρ_max_ = 0.12 e Å^−3^
Δρ_min_ = −0.17 e Å^−3^

### 

Data collection: *APEX2* (Bruker, 2004 ▶); cell refinement: *APEX2* and *SAINT* (Bruker, 2004 ▶); data reduction: *SAINT* and *XPREP* (Bruker, 2004 ▶); program(s) used to solve structure: *SHELXS97* (Sheldrick, 2008 ▶); program(s) used to refine structure: *SHELXL97* (Sheldrick, 2008 ▶); molecular graphics: *ORTEP-3* (Farrugia, 1997 ▶); software used to prepare material for publication: *SHELXL97* and *PLATON* (Spek, 2009 ▶)’.

## Supplementary Material

Crystal structure: contains datablocks global, I. DOI: 10.1107/S1600536809006023/bt2875sup1.cif

Structure factors: contains datablocks I. DOI: 10.1107/S1600536809006023/bt2875Isup2.hkl

Additional supplementary materials:  crystallographic information; 3D view; checkCIF report

## Figures and Tables

**Table 1:** Hydrogen-bond geometry (Å, °)

*D*—H⋯*A*	*D*—H	H⋯*A*	*D*⋯*A*	*D*—H⋯*A*
C11—H11⋯O3^i^	0.93	2.54	3.395 (2)	153
C18—H18⋯O4^ii^	0.93	2.44	3.259 (2)	148
N1—H1⋯O4^ii^	0.86	2.36	3.078 (2)	141
N1—H1⋯O4	0.86	2.02	2.670 (2)	132

Symmetry codes: (i) 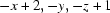; (ii) 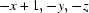.

**Fig. 1.**
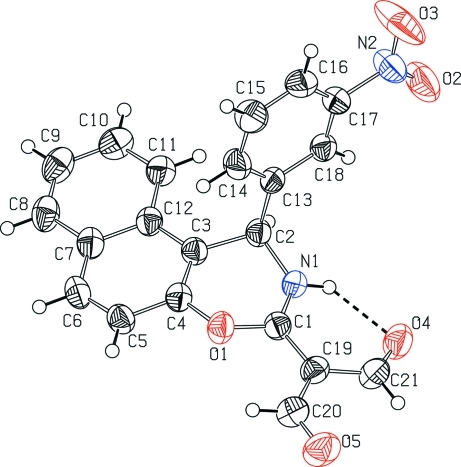
The molecular structure of (I), showing the atom-numbering scheme for (I). Displacement ellipsoids are drawn at the 50% probability level.

**Fig. 2.**
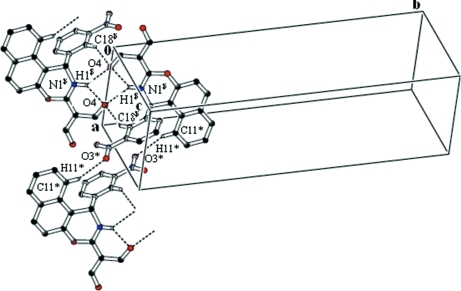
The crystal packing of compound (I), showing the R22(12), R22(20) and R22(18) rings. Hydrogen bonding is shown as dashed lines. H atoms not involved in the hydrogen bonding have been omitted for clarity. [Symmetry codes: (*)-x + 2, -y, -z + 1, ($)-x + 1, -y, -z]

## supplementary crystallographic information

### Comment

A view of the title compound with the atom-numbering scheme is shown in Fig. 1.   The bond lengths C1—O1 and C1—N1 in the oxazine ring are 1.344 (2) and 1.307 (2)Å respectively.

The six membered   oxazine moiety is not planar, having a total puckering amplitude, Q_T_ of 0.329 (2) Å and adopting a boat conformation [θ = 81.8 (1) and φ = 171.8 (2)°] (Cremer & Pople, 1975), and the lowest displacement asymmetry parameters Δ_S_(O1) is 8.0 (1)° (Nardelli, 1983). The dihedral angle between the naphthalene and benzene ring is 89.2 (1)°.

The crystal structure of the title compound is characterized by bifurcated hydrogen bonds between the amine and aldehyde groups (Table 1 and Fig. 2). One interaction is an intramolecular N—H···O hydrogen bond between donor atom N1 and acceptor atom O4, described by the graph-set motif S(6) (Bernstein *et al.*, 1995). Atom N1 in the molecule at (*x*, *y*, *z*) also acts as a hydrogen-bond donor *via* atom H1 to atom O4 in the molecule at (1 - *x*, -*y*, -*z*). This intermolecular hydrogen bond links the molecules into dimers described by a graph-set motif *R*^2^_2_(12). The structure also contains C—H···O intermolecular interactions. Atom C11 and C18 in the molecule at (*x*, *y*, *z*) acts as a hydrogen-bond donor *via* atom H11 and H18 to atom O3 and O4 in the molecule at (2 - *x*, -*y*, 1 - *z*). This intermolecular hydrogen bond links the molecules into dimers with a cyclic *R*^2^_2_(20) and *R*^2^_2_(18) ring system, respectively.

### Experimental

To the solution containing *N*-[(3-Nitro-phenyl)-(2-hydroxy-napthalen-1-yl) -methyl]-acetamide (10 mmol) dissolved in DMF (12 equiv), POCl3 (8 eqiuv) was added slowly dropwise (around 15 min) at 273°K, then the reaction mixture was allowed to reach room temperature. The reaction mixture was stirred at 363°K for about 3.5 h. Afer completion of the reaction, it was allowed to cool to room temperature. Then it was poured into crushed ice and refrigerated overnight. The solution was neutralized with sodium acetate and the crude compound was extracted with dichloromethane (3 × 50 ml) and washed with water (3 × 25 ml). Organic layer was dried over anhydrous sodium sulfate and concentrated under reduced pressure. The crude product was purified through column chromatography using ethyl acetate: petroleum ether (1:1) as eluent. Single crystals of the title compound suitable for X-ray diffraction were obtained by slow evaporation of a solution in Ethanol.

### Refinement

All H atoms were positioned geometrically, with N—H = 0.86 and C—H = 0.93 and 0.98 Å aromatic and methylene H, respectively, and constrained to ride on their parent atoms, with *U*_iso_(H) = *xU*_eq_(C, N), where *x* = 1.2 for all H atoms.

### Crystal data

**Table:**

C_21_H_14_N_2_O_5_	*F*(000) = 776
*M**_r_* = 374.34	*D*_x_ = 1.442 Mg m^−^^3^
Monoclinic, *P*2_1_/*n*	Mo *K*α radiation, λ = 0.71073 Å
Hall symbol: -P 2yn	Cell parameters from 2334 reflections
*a* = 7.9191 (4) Å	θ = 2.5–25°
*b* = 23.3876 (11) Å	µ = 0.11 mm^−^^1^
*c* = 9.6199 (5) Å	*T* = 293 K
β = 104.632 (2)°	Block, colourless
*V* = 1723.91 (15) Å^3^	0.33 × 0.27 × 0.25 mm
*Z* = 4	

### Data collection

**Table:**

'Bruker Kappa APEXII CCD diffractometer'	3023 independent reflections
Radiation source: fine-focus sealed tube	2334 reflections with *I* > 2σ(*I*)
graphite	*R*_int_ = 0.032
ω and φ scan	θ_max_ = 24.9°, θ_min_ = 2.8°
Absorption correction: multi-scan (*SADABS*; Bruker, 2004)	*h* = −9→9
*T*_min_ = 0.897, *T*_max_ = 0.974	*k* = −27→27
16263 measured reflections	*l* = −11→11

### Refinement

**Table:**

Refinement on *F*^2^	Primary atom site location: structure-invariant direct methods
Least-squares matrix: full	Secondary atom site location: difference Fourier map
*R*[*F*^2^ > 2σ(*F*^2^)] = 0.036	Hydrogen site location: inferred from neighbouring sites
*wR*(*F*^2^) = 0.102	H-atom parameters constrained
*S* = 1.00	*w* = 1/[σ^2^(*F*_o_^2^) + (0.0509*P*)^2^ + 0.352*P*] where *P* = (*F*_o_^2^ + 2*F*_c_^2^)/3
3023 reflections	(Δ/σ)_max_ < 0.001
253 parameters	Δρ_max_ = 0.12 e Å^−^^3^
0 restraints	Δρ_min_ = −0.17 e Å^−^^3^

### Special details

**Table:**

Geometry. All e.s.d.'s (except the e.s.d. in the dihedral angle between two l.s. planes) are estimated using the full covariance matrix. The cell e.s.d.'s are taken into account individually in the estimation of e.s.d.'s in distances, angles and torsion angles; correlations between e.s.d.'s in cell parameters are only used when they are defined by crystal symmetry. An approximate (isotropic) treatment of cell e.s.d.'s is used for estimating e.s.d.'s involving l.s. planes.
Refinement. Refinement of *F*^2^ against ALL reflections. The weighted *R*-factor *wR* and goodness of fit *S* are based on *F*^2^, conventional *R*-factors *R* are based on *F*, with *F* set to zero for negative *F*^2^. The threshold expression of *F*^2^ > σ(*F*^2^) is used only for calculating *R*-factors(gt) *etc*. and is not relevant to the choice of reflections for refinement. *R*-factors based on *F*^2^ are statistically about twice as large as those based on *F*, and *R*- factors based on ALL data will be even larger.

### Fractional atomic coordinates and isotropic or equivalent isotropic displacement parameters (Å^2^)

**Table:**

	*x*	*y*	*z*	*U*_iso_*/*U*_eq_	
O1	0.30114 (13)	0.16841 (5)	0.13932 (12)	0.0486 (3)	
O2	0.8553 (2)	−0.07387 (6)	0.43720 (18)	0.0878 (5)	
O3	0.8486 (3)	−0.07086 (8)	0.6565 (2)	0.1195 (7)	
O4	0.30819 (17)	0.00916 (5)	−0.05620 (14)	0.0638 (4)	
O5	−0.14205 (16)	0.09772 (6)	−0.06754 (16)	0.0718 (4)	
N1	0.47317 (16)	0.09268 (5)	0.11444 (13)	0.0418 (3)	
H1	0.4847	0.0619	0.0690	0.050*	
N2	0.8208 (2)	−0.04987 (7)	0.53794 (19)	0.0637 (4)	
C1	0.3180 (2)	0.11583 (6)	0.08808 (16)	0.0401 (4)	
C2	0.62913 (19)	0.11553 (6)	0.21612 (16)	0.0388 (4)	
H2	0.7310	0.1063	0.1799	0.047*	
C3	0.61165 (19)	0.17956 (6)	0.21877 (16)	0.0395 (4)	
C4	0.45014 (19)	0.20287 (6)	0.18043 (17)	0.0412 (4)	
C5	0.4178 (2)	0.26167 (7)	0.17688 (17)	0.0464 (4)	
H5	0.3042	0.2757	0.1503	0.056*	
C6	0.5556 (2)	0.29776 (7)	0.21305 (18)	0.0485 (4)	
H6	0.5363	0.3370	0.2088	0.058*	
C7	0.7285 (2)	0.27682 (7)	0.25716 (17)	0.0439 (4)	
C8	0.8737 (2)	0.31383 (8)	0.29883 (19)	0.0529 (4)	
H8	0.8562	0.3532	0.2938	0.063*	
C9	1.0376 (2)	0.29305 (8)	0.3459 (2)	0.0576 (5)	
H9	1.1313	0.3181	0.3739	0.069*	
C10	1.0666 (2)	0.23391 (8)	0.35255 (19)	0.0555 (5)	
H10	1.1796	0.2199	0.3856	0.067*	
C11	0.9305 (2)	0.19657 (7)	0.31089 (17)	0.0474 (4)	
H11	0.9518	0.1574	0.3150	0.057*	
C12	0.7579 (2)	0.21687 (7)	0.26167 (16)	0.0402 (4)	
C13	0.65335 (18)	0.08758 (6)	0.36265 (16)	0.0370 (3)	
C14	0.6049 (2)	0.11392 (7)	0.47526 (18)	0.0504 (4)	
H14	0.5557	0.1503	0.4621	0.061*	
C15	0.6281 (2)	0.08734 (8)	0.60702 (19)	0.0590 (5)	
H15	0.5947	0.1060	0.6812	0.071*	
C16	0.6998 (2)	0.03369 (8)	0.62976 (18)	0.0510 (4)	
H16	0.7172	0.0157	0.7184	0.061*	
C17	0.7447 (2)	0.00769 (7)	0.51666 (18)	0.0441 (4)	
C18	0.72429 (19)	0.03325 (7)	0.38466 (17)	0.0410 (4)	
H18	0.7578	0.0142	0.3110	0.049*	
C19	0.1647 (2)	0.08978 (7)	0.00800 (16)	0.0425 (4)	
C20	−0.0008 (2)	0.11766 (8)	−0.00797 (18)	0.0525 (4)	
H20	0.0004	0.1542	0.0307	0.063*	
C21	0.1741 (2)	0.03622 (8)	−0.05856 (18)	0.0529 (4)	
H21	0.0691	0.0200	−0.1087	0.063*	

### Atomic displacement parameters (Å^2^)

**Table:**

	*U*^11^	*U*^22^	*U*^33^	*U*^12^	*U*^13^	*U*^23^
O1	0.0428 (6)	0.0418 (7)	0.0611 (7)	−0.0005 (5)	0.0128 (5)	−0.0115 (5)
O2	0.1298 (13)	0.0574 (9)	0.0767 (11)	0.0390 (9)	0.0270 (10)	0.0026 (8)
O3	0.1869 (19)	0.1002 (13)	0.0839 (12)	0.0799 (13)	0.0572 (12)	0.0540 (10)
O4	0.0610 (8)	0.0556 (8)	0.0703 (9)	0.0025 (6)	0.0082 (6)	−0.0219 (6)
O5	0.0463 (7)	0.0827 (10)	0.0801 (10)	−0.0028 (7)	0.0041 (7)	−0.0112 (8)
N1	0.0474 (8)	0.0357 (7)	0.0391 (7)	0.0028 (6)	0.0050 (6)	−0.0071 (6)
N2	0.0758 (10)	0.0516 (9)	0.0642 (11)	0.0201 (8)	0.0189 (8)	0.0122 (8)
C1	0.0477 (9)	0.0381 (9)	0.0355 (8)	0.0008 (7)	0.0123 (7)	−0.0007 (7)
C2	0.0400 (8)	0.0348 (8)	0.0408 (9)	0.0007 (6)	0.0089 (7)	−0.0045 (7)
C3	0.0466 (9)	0.0352 (8)	0.0369 (8)	0.0012 (7)	0.0111 (7)	−0.0008 (7)
C4	0.0447 (9)	0.0375 (9)	0.0419 (9)	−0.0009 (7)	0.0116 (7)	−0.0032 (7)
C5	0.0504 (9)	0.0402 (9)	0.0490 (10)	0.0084 (8)	0.0134 (8)	−0.0002 (7)
C6	0.0645 (11)	0.0330 (9)	0.0511 (10)	0.0038 (8)	0.0202 (8)	0.0013 (7)
C7	0.0582 (10)	0.0380 (9)	0.0392 (9)	−0.0041 (7)	0.0191 (7)	−0.0002 (7)
C8	0.0672 (12)	0.0434 (10)	0.0530 (11)	−0.0129 (9)	0.0245 (9)	−0.0026 (8)
C9	0.0598 (11)	0.0612 (12)	0.0553 (11)	−0.0230 (9)	0.0211 (9)	−0.0057 (9)
C10	0.0486 (10)	0.0660 (12)	0.0531 (11)	−0.0078 (9)	0.0150 (8)	−0.0017 (9)
C11	0.0491 (9)	0.0467 (10)	0.0470 (10)	−0.0016 (8)	0.0133 (8)	0.0000 (8)
C12	0.0482 (9)	0.0386 (9)	0.0354 (8)	−0.0028 (7)	0.0136 (7)	−0.0010 (7)
C13	0.0338 (7)	0.0342 (8)	0.0404 (8)	−0.0017 (6)	0.0044 (6)	−0.0046 (7)
C14	0.0612 (11)	0.0413 (10)	0.0485 (10)	0.0106 (8)	0.0133 (8)	−0.0042 (8)
C15	0.0745 (12)	0.0609 (12)	0.0441 (11)	0.0126 (10)	0.0197 (9)	−0.0063 (9)
C16	0.0554 (10)	0.0559 (11)	0.0391 (9)	0.0020 (8)	0.0071 (7)	0.0031 (8)
C17	0.0431 (9)	0.0396 (9)	0.0466 (10)	0.0042 (7)	0.0056 (7)	0.0025 (7)
C18	0.0415 (8)	0.0389 (9)	0.0417 (9)	0.0028 (7)	0.0088 (7)	−0.0055 (7)
C19	0.0448 (9)	0.0458 (10)	0.0362 (8)	−0.0034 (7)	0.0087 (7)	−0.0027 (7)
C20	0.0488 (10)	0.0591 (11)	0.0482 (10)	−0.0017 (8)	0.0094 (8)	−0.0038 (8)
C21	0.0511 (10)	0.0563 (11)	0.0482 (10)	−0.0069 (9)	0.0068 (8)	−0.0092 (8)

### Geometric parameters (Å, °)

**Table:**

O1—C1	1.3440 (18)	C8—C9	1.352 (3)
O1—C4	1.4009 (18)	C8—H8	0.9300
O2—N2	1.209 (2)	C9—C10	1.401 (3)
O3—N2	1.210 (2)	C9—H9	0.9300
O4—C21	1.232 (2)	C10—C11	1.366 (2)
O5—C20	1.214 (2)	C10—H10	0.9300
N1—C1	1.3075 (19)	C11—C12	1.411 (2)
N1—C2	1.4689 (19)	C11—H11	0.9300
N1—H1	0.8600	C13—C14	1.382 (2)
N2—C17	1.468 (2)	C13—C18	1.384 (2)
C1—C19	1.402 (2)	C14—C15	1.382 (3)
C2—C3	1.505 (2)	C14—H14	0.9300
C2—C13	1.521 (2)	C15—C16	1.372 (2)
C2—H2	0.9800	C15—H15	0.9300
C3—C4	1.353 (2)	C16—C17	1.370 (2)
C3—C12	1.425 (2)	C16—H16	0.9300
C4—C5	1.398 (2)	C17—C18	1.376 (2)
C5—C6	1.354 (2)	C18—H18	0.9300
C5—H5	0.9300	C19—C21	1.417 (2)
C6—C7	1.414 (2)	C19—C20	1.437 (2)
C6—H6	0.9300	C20—H20	0.9300
C7—C8	1.414 (2)	C21—H21	0.9300
C7—C12	1.420 (2)		
			
C1—O1—C4	118.37 (12)	C11—C10—C9	120.65 (17)
C1—N1—C2	124.71 (13)	C11—C10—H10	119.7
C1—N1—H1	117.6	C9—C10—H10	119.7
C2—N1—H1	117.6	C10—C11—C12	120.58 (16)
O2—N2—O3	122.89 (17)	C10—C11—H11	119.7
O2—N2—C17	118.80 (16)	C12—C11—H11	119.7
O3—N2—C17	118.31 (17)	C11—C12—C7	118.71 (14)
N1—C1—O1	118.90 (14)	C11—C12—C3	122.58 (14)
N1—C1—C19	124.59 (14)	C7—C12—C3	118.70 (14)
O1—C1—C19	116.51 (13)	C14—C13—C18	118.15 (15)
N1—C2—C3	107.93 (12)	C14—C13—C2	122.67 (14)
N1—C2—C13	110.41 (12)	C18—C13—C2	119.18 (13)
C3—C2—C13	113.73 (12)	C13—C14—C15	121.30 (16)
N1—C2—H2	108.2	C13—C14—H14	119.3
C3—C2—H2	108.2	C15—C14—H14	119.3
C13—C2—H2	108.2	C16—C15—C14	120.76 (16)
C4—C3—C12	118.39 (14)	C16—C15—H15	119.6
C4—C3—C2	118.75 (13)	C14—C15—H15	119.6
C12—C3—C2	122.86 (13)	C17—C16—C15	117.36 (16)
C3—C4—O1	121.07 (13)	C17—C16—H16	121.3
C3—C4—C5	123.91 (15)	C15—C16—H16	121.3
O1—C4—C5	115.02 (13)	C16—C17—C18	123.15 (15)
C6—C5—C4	118.41 (15)	C16—C17—N2	118.43 (15)
C6—C5—H5	120.8	C18—C17—N2	118.41 (15)
C4—C5—H5	120.8	C17—C18—C13	119.26 (14)
C5—C6—C7	121.19 (15)	C17—C18—H18	120.4
C5—C6—H6	119.4	C13—C18—H18	120.4
C7—C6—H6	119.4	C1—C19—C21	119.67 (15)
C6—C7—C8	121.97 (15)	C1—C19—C20	120.10 (15)
C6—C7—C12	119.34 (15)	C21—C19—C20	120.22 (15)
C8—C7—C12	118.69 (15)	O5—C20—C19	125.67 (18)
C9—C8—C7	121.21 (17)	O5—C20—H20	117.2
C9—C8—H8	119.4	C19—C20—H20	117.2
C7—C8—H8	119.4	O4—C21—C19	126.11 (16)
C8—C9—C10	120.15 (16)	O4—C21—H21	116.9
C8—C9—H9	119.9	C19—C21—H21	116.9
C10—C9—H9	119.9		
			
C2—N1—C1—O1	10.5 (2)	C8—C7—C12—C3	−179.59 (14)
C2—N1—C1—C19	−169.97 (14)	C4—C3—C12—C11	176.48 (15)
C4—O1—C1—N1	18.7 (2)	C2—C3—C12—C11	−2.7 (2)
C4—O1—C1—C19	−160.81 (13)	C4—C3—C12—C7	−2.3 (2)
C1—N1—C2—C3	−31.1 (2)	C2—C3—C12—C7	178.44 (14)
C1—N1—C2—C13	93.82 (17)	N1—C2—C13—C14	−100.41 (16)
N1—C2—C3—C4	24.17 (19)	C3—C2—C13—C14	21.1 (2)
C13—C2—C3—C4	−98.69 (16)	N1—C2—C13—C18	78.61 (16)
N1—C2—C3—C12	−156.62 (13)	C3—C2—C13—C18	−159.90 (13)
C13—C2—C3—C12	80.52 (17)	C18—C13—C14—C15	0.8 (2)
C12—C3—C4—O1	−179.04 (13)	C2—C13—C14—C15	179.79 (16)
C2—C3—C4—O1	0.2 (2)	C13—C14—C15—C16	−0.2 (3)
C12—C3—C4—C5	1.6 (2)	C14—C15—C16—C17	−0.8 (3)
C2—C3—C4—C5	−179.12 (15)	C15—C16—C17—C18	1.3 (3)
C1—O1—C4—C3	−24.0 (2)	C15—C16—C17—N2	−179.66 (15)
C1—O1—C4—C5	155.39 (14)	O2—N2—C17—C16	177.49 (18)
C3—C4—C5—C6	0.4 (2)	O3—N2—C17—C16	−3.6 (3)
O1—C4—C5—C6	−178.97 (14)	O2—N2—C17—C18	−3.4 (3)
C4—C5—C6—C7	−1.7 (2)	O3—N2—C17—C18	175.53 (19)
C5—C6—C7—C8	−178.35 (16)	C16—C17—C18—C13	−0.7 (2)
C5—C6—C7—C12	0.9 (2)	N2—C17—C18—C13	−179.79 (14)
C6—C7—C8—C9	177.48 (16)	C14—C13—C18—C17	−0.3 (2)
C12—C7—C8—C9	−1.8 (2)	C2—C13—C18—C17	−179.37 (13)
C7—C8—C9—C10	0.8 (3)	N1—C1—C19—C21	−4.3 (2)
C8—C9—C10—C11	0.4 (3)	O1—C1—C19—C21	175.26 (14)
C9—C10—C11—C12	−0.6 (3)	N1—C1—C19—C20	176.56 (15)
C10—C11—C12—C7	−0.4 (2)	O1—C1—C19—C20	−3.9 (2)
C10—C11—C12—C3	−179.22 (15)	C1—C19—C20—O5	−175.50 (17)
C6—C7—C12—C11	−177.72 (14)	C21—C19—C20—O5	5.3 (3)
C8—C7—C12—C11	1.5 (2)	C1—C19—C21—O4	−1.5 (3)
C6—C7—C12—C3	1.2 (2)	C20—C19—C21—O4	177.67 (17)

### Hydrogen-bond geometry (Å, °)

**Table:**

*D*—H···*A*	*D*—H	H···*A*	*D*···*A*	*D*—H···*A*
C11—H11···O3^i^	0.93	2.54	3.395 (2)	153
C18—H18···O4^ii^	0.93	2.44	3.259 (2)	148
N1—H1···O4^ii^	0.86	2.36	3.078 (2)	141
N1—H1···O4	0.86	2.02	2.670 (2)	132
C20—H20···O1	0.93	2.37	2.724 (2)	102

Symmetry codes: (i) −*x*+2, −*y*, −*z*+1; (ii) −*x*+1, −*y*, −*z*.
